# The Motivating Function of Healthcare Professional in eHealth and mHealth Interventions for Type 2 Diabetes Patients and the Mediating Role of Patient Engagement

**DOI:** 10.1155/2016/2974521

**Published:** 2016-01-05

**Authors:** Guendalina Graffigna, Serena Barello, Andrea Bonanomi, Julia Menichetti

**Affiliations:** ^1^Department of Psychology, Università Cattolica del Sacro Cuore, Largo A. Gemelli 1, 20123 Milan, Italy; ^2^Department of Statistical Sciences, Università Cattolica del Sacro Cuore, Largo A. Gemelli 1, 20123 Milan, Italy

## Abstract

eHealth and mHealth interventions for type 2 diabetes are emerging as useful strategies to accomplish the goal of a high functioning integrated care system. However, mHealth and eHealth interventions in order to be successful need the clear endorsement from the healthcare professionals. This cross-sectional study included a sample of 93 Italian-speaking type 2 diabetes patients and demonstrated the role of the perceived ability of healthcare professionals to motivate patients' initiative in improving the level of their engagement and activation in type 2 diabetes self-management. The level of type 2 diabetes patients' activation resulted also in being a direct precursor of their attitude to the use of mHealth and eHealth. Furthermore, patient engagement has been demonstrated to be a mediator of the relationship between the perceived ability of healthcare professionals in motivating type 2 diabetes patients and patients' activation. Finally, type 2 diabetes patients adherence did not result in being a direct consequence of the frequency of mHealth and eHealth use. Patient adherence appeared to be directly influenced by the level of perceived healthcare professionals ability of motivating patients' autonomy. These results offer important insights into the psychosocial and organizational elements that impact on type 2 diabetes patients' activation in self-management and on their willingness to use mHealth and eHealth devices.

## 1. Introduction

Diabetes currently constitutes a large and growing clinical problem, and its costs for society are high and are escalating. Worldwide, estimated 387 million adults are living with diabetes, and this number is projected to increase to 592 million by 2035 [1–3]. Effective prevention strategies are, therefore, crucial to slow the diabetes tide and its burden. Nearly 9 out of 10 new diabetes cases are type 2 diabetes, characterized by a gradual increase in glycemia [1]; obesity and physical inactivity are some of the most common risk factors [2].

Since type 2 diabetes requires long-term treatment, over the past 20 years the responsibility for the care of people affected by this condition has shifted away from hospitals to primary care settings. The long-term management of chronic conditions requires a revision of classical models of care in order to guarantee positive care outcomes [4] and enhance patient's quality of life [5]. To address this requirement and to manage the patients' care, a more effective synergy between healthcare organizations and territorial services is required [6–8]. Chronic conditions, such as type 2 diabetes, need long-term approach to care, which imply a higher synergy and service integration “outside” of the institutional boundaries of hospitals [9–11]. Thus healthcare organizations not only are concerned with the long-term management of type 2 diabetes patient but also are claimed to redesign their organizational models in accordance with local resources and demands of care. This requires a better integration with the resources (formal and informal; expert and lay) that are present in the territories [12, 13].

Integrated care organizational models are currently envisaged as the potential solution to improve quality and sustainability of healthcare services, particularly when the management of chronic condition (such as type 2 diabetes) is concerned. However, to achieve the goal of an integrated system of care, the role of the patient, as main actor of such a process, needs to be questioned [14]. In order to guarantee the fruitful collaboration and dialogue between the lay territory of reference for the patient and his/her reference healthcare provider, type 2 diabetes patients need to be helped in enacting an active and cocreative role along their process of care, moving from the traditional passive position of recipients of care to the one of the real engaged consumers in the design and delivery of healthcare services [15–18]. Type 2 diabetes patients' engagement is regarded as a key factor to improve the quality and the sustainability of healthcare services [15, 17, 19]. Previous studies have shown how an engaged patient is more likely keen to act improved health behaviors [20], to have better clinical outcomes [21], to perceive a better quality of life [22], and to be more satisfied with their relationship with the healthcare system [23]. Furthermore, empirical researches have demonstrated how patient engagement may contribute to a reduction of healthcare costs and to better economically sustainable organizational processes [24, 25].

In such a frame, eHealth and mHealth interventions are emerging as a useful strategy to accomplish the goal of a better integrated system of care [11, 26, 27]. As technology-based interventions are becoming regular part of the health care environment, viewing these tools in light of the skills (knowledge and behaviors) required for patients to successfully use them becomes essential if the power of eHealth and mHealth is to be leveraged to deliver health care effectively. As a consequence, promoting patient's eHealth literacy, defined as the ability to seek, find, understand, and appraise health information from electronic sources and apply the knowledge gained to addressing or solving a health problem [28], becomes a priority to enhance the continuity of care. Indeed, eHealth and mHealth offer continuous monitoring of clinical parameters, allowing the “on-demand” communication with the reference healthcare professionals, and, consequently, they are able of empowering the patient in the self-management of the disease condition and his/her therapy [29, 30]. A systematic review showed a positive impact of mHealth on patient engagement in the management of chronic diseases [31]: diabetic patients who transferred daily glucose readings to physicians using a telematics system and received telephone medication regimen feedback improved their clinical outcomes and presented a better glycemic control [32]. Likewise, the use of text message interventions, such as reminders and updates through SMS, ensured a greater adherence to prescription and improved clinical outcomes [33]. Furthermore, studies confirmed the effectiveness of mHealth interventions in modifying type 2 diabetes patients lifestyles, especially those related to dietary behaviors and physical activity, by facilitating diabetes self-management processes outside the clinical setting [34–36].

However, mHealth and eHealth interventions in order to be successful need the clear endorsement from the healthcare system. Particularly, the reference healthcare professionals are the key actors, from the patients' perspective, that can legitimize the intervention process and can motivate type 2 diabetes patients in being compliant with mHealth and eHealth [37]. This underlines the role of healthcare organizational and professional cultures in enhancing or inhibiting the effectiveness of mHealth and eHealth interventions in managing type 2 diabetes. More attention is needed to explore how innovation through the introduction of new health technologies can be integrated in the systems of symbols, practices, and power relationships already existent in healthcare organizations [38]. Thus, the enabling role of healthcare professionals in the eHealth and mHealth interventions for type 2 diabetes needs to be further considered as a fundamental ingredient for their clinical success. Healthcare professionals should sustain type 2 diabetes patients' autonomy in care management and thus their motivation to adhere to the mHealth and eHealth intervention.

Based on these premises, the present study, carried out on a sample of Italian type 2 diabetes patients, was aimed at verifying the following hypotheses:

1. The perceived ability of the healthcare professionals to support patients' autonomy influences the level of patients' engagement towards their care management.
2. The perceived ability of the healthcare professionals to support patients' autonomy influences the level of patients' activation towards their care management.
3. The levels of patients engagement mediate the association between the perceived ability of healthcare professionals to support patients' autonomy and the level of patients' activation.
4. A higher level of activation is associated with a higher use of mHealth and eHealth technologies to seek information for managing type 2 diabetes care.
5. A higher level of use of mHealth and eHealth technologies to seek information for managing type 2 diabetes care is associated with a higher patients' adherence to type 2 diabetes care.

## 2. Materials and Methods

### 2.1. Recruitment and Data Collection

This cross-sectional quantitative study included a sample of 93 Italian-speaking type 2 diabetes patients and was conducted on the basis of a structured questionnaire including validated measures (see Section 2.2) to assess the causal relations among the constructs under analysis (see research hypotheses stated above). Patients were recruited through the online panel provided by Research Now (http://www.researchnow.com/en-US.aspx). The panel covers a wide range of chronic conditions and counts more than 6.5 million registered subjects worldwide. Subjects belonging to the panel are carefully screened for authenticity and legitimacy via digital fingerprint and geo-IP-validation from the provider. All panelists are profiled on the basis of their sociodemographic, clinical, and lifestyle characteristics. The panel is certified to be statistically representative of all the covered populations. In our study, in order to guarantee data quality, respondents were asked to confirm their demographics (i.e., sex, date and place of birth, ethnicity, nationality, educational level, and place of residency) and clinical condition previously collected by the panel. To be included in our study, patients belonging to the panel had to be Italian, affected by type 2 diabetes, aged over 18 years, and of both genders. Patients with dementia, cognitive impairments, active psychiatric disorders, blindness, deafness, or insufficient Italian language skills to meaningfully answer the questions or without informed consent were excluded from this study. All participants gave written informed consent before being enrolled in the study. Patients completed the study questionnaire between October and December 2014. Ethic approval was attained from the Ethics Committee of the Università Cattolica del Sacro Cuore, Milan (Italy).

### 2.2. Measures

*Patient Health Engagement Scale (PHE-S)* developed by Graffigna and colleagues [39] is a measure of patient engagement that is grounded in rigorous conceptualization and appropriate psychometric methods. The scale consists of 5 ordinal items and was developed based on the authors' conceptual model of patient engagement (PHE-model), which features four positions along a continuum of engagement (i.e., blackout; arousal; adhesion; eudaimonic project). These engagement positions result from the conjoint cognitive (thinking), emotional (feeling), and conative (acting) enactment of individuals toward their health management [15].

*Patient Activation Measure (PAM)* developed by Hibbard and colleagues [40], the 13-item Patient Activation Measure, is an interval-level, unidimensional Guttman-like measure that contains items measuring self-assessed knowledge about chronic conditions, beliefs about illness and medical care, and self-efficacy for self-care. The PAM focused on physical conditions, and it was designed to measure activation as a broad construct. In the present study, we used the Italian validated version of the PAM [41].

*Morisky Medication Adherence Scale (MMAS-4)*. Medication-taking behavior was assessed using the 4-item Morisky Medication Adherence Scale. This simple 4-question survey assesses the likelihood of patients taking their drug therapy as prescribed. The items measure the degree to which the patients self-report nonadherence to prescribed medication due to forgetting, carelessness, stopping the drug when feeling better or stopping the drug when feeling worse. In the present study, we used the Italian validated version of the MMAS-4 [42].

*Health Care Climate Questionnaire (HCCQ)*. This scale assesses patients' perceptions of the ability of the healthcare professionals in supporting their autonomy (versus “controllingness”) and in motivating their initiative in care management. The HCCQ consists of 15 items on a seven-point Likert scale ranging from* strongly disagree* to* strongly agree*. The scale was firstly developed and validated on the diabetic population by Williams and colleagues [43, 44].

*Demographic characteristics* included age (<60; ≥60); gender (male or female); education (elementary school, junior high school, high school, college education, Ph.D. degree, or M.S. degree); occupational status (employed, retired, housewife, student, unemployed, or other); marital status (never married, married, divorced, or widowed).

*Frequency of mHealth/eHealth Use*. An ad hoc item was developed to assess patients' behaviors concerning the use of mHealth and eHealth technologies to seek information for managing type 2 diabetes care (i.e., “*I usually use internet or mobile devices to seek information for managing my care*”). The item has 7 response options on a Likert scale (never, almost never, occasionally, sometimes, often, almost always, or always).

### 2.3. Data Analysis

Data analysis was conducted in four steps. In the first step of analysis, descriptive analyses were conducted, with particular reference to sociodemographic characteristics of the sample. Furthermore, descriptive statistics were provided regarding the use of mHealth and eHealth technologies to seek information for managing type 2 diabetes care.

In the second step of the analysis, the psychometric properties of the instruments were assessed in terms of reliability by using Cronbach's alpha for metric variables or ordinal alpha via Empirical Copula for ordinal variables [45]. A Cronbach or ordinal alpha higher than 0.7 was considered acceptable.

In the third step of analysis, correlations between all the considered variables were calculated. Since every instrument produces a metric score, the linear correlation coefficient *r* was calculated and evaluated with a significance test.

In the last step, a Structural Equation Model with observed variables using ML estimation method was implemented [46], in order to evaluate the relationships between the considered variables and to explore the theoretical hypothesized model (see the 5 hypotheses stated above). In the model we considered* HCCQ* as an exogenous variable and mediator (*PHE*-S) and dependent variables (*PAM, MMAS-4, *and* frequency of mHealth/eHealth use*) as endogenous variables. The goodness-of-fit indexes were examined through Chi square test, RMSEA, CFI, and SRMR, particularly suitable for both large and small samples. Models with acceptable fit presented nonsignificant Chi square value, RMSEA < 0.08 CFI > 0.90 and SRMR < 0.08 [47]. To improve the goodness-of-fit, modification indices were considered.

### 2.4. Ethical Concerns

The study received approval from the Università Cattolica del Sacro Cuore Ethics Committee. Patients consented to participate in the study, and they were allowed to withdraw from the study whenever they wanted. The data were collected anonymously and analyzed in an aggregated way.

## 3. Results

Overall, 93 patients were invited to participate in the study and completely answered the questionnaire for the analysis. All patients (29 females) completed the survey, mean age of 58.3 (±12.4) years with a mean disease duration of almost 11 years. Sociodemographic and psychometric characteristics are summarized in Table 1. Mean, standard deviation (unless otherwise indicated), and a suitable reliability index (Cronbach's alpha or ordinal alpha via Empirical Copula) are reported for all the psychometric measures considered. All the psychometric measures presented a good or excellent reliability, with a Cronbach's or ordinal alpha ranged from 0.81 to 0.93.

Table 2 reports the distribution of the ad hoc item (*frequency of mHealth/eHealth use*), created to assess patients' behaviors concerning the use of mHealth and eHealth technologies to seek information for managing type 2 diabetes care (i.e., “*I usually use internet or mobile devices to seek information for managing my care*”). Table 2 shows that much more than 50% of our sample used regularly (i.e., often, very often, or always) mHealth or eHealth technologies to seek for information for managing their type 2 diabetes care. Only 20% of the sample did not regularly use such technologies.

In Table 3 linear correlation coefficients between the considered psychometric variables are reported.

*HCCQ* presented a significant correlation with all the measures: a positive correlation with* PHE-S, PAM,* and* frequency of mHealth/eHealth use* and a negative correlation with* MMAS-4* were detected.* PHE-S* showed a significant direct correlation with* HCCQ* and* PAM*, while it had no significant correlation with* MMAS-4* and* frequency of mHealth/eHealth use*.* PAM* had a significant direct correlation with all the measures except from* MMAS-4*:* PAM* and* MMAS-4* were negatively correlated.* Frequency of mHealth/eHealth use* significantly only depended on* HCCQ* and* PAM*.

Considering the five hypotheses to be tested in the study and the detected correlations between the psychometric measures and the* frequency of mHealth/eHealth use*, a Structural Equation Model was implemented.

**Figure 1:**
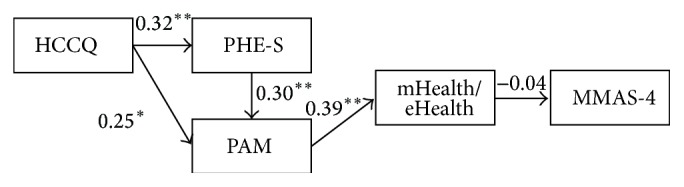
Structural Equation Model 1.

Relationships between patients' perceptions of the ability of the healthcare professionals in supporting their autonomy (*HCCQ*), patients' engagement (*PHE-S*), patient's activation (*PAM*), medication adherence (*MMAS-4*), and the* frequency of mHealth/eHealth use* were tested.  Figure 1 shows the explanatory model of the hypotheses we wanted to verify.

The model showed an exogenous observed variable (*HCCQ*), four endogenous observed variables (PHE-S, PAM,* frequency of mHealth/eHealth use,* and* MMAS-4*). The* PHE-S* mediates the relationship between* HCCQ* and* PAM*.

The model fit was deemed to be not acceptable (*χ* ^2^(5) = 15.50, *p* < 0.01; CFI = 0.59; RMSEA = 0.15). Almost all the paths were found to be significant (^*∗∗*^ *p* < 0.01, ^*∗*^ *p* < 0.05), except the path between* frequency of mHealth/eHealth use* and* MMAS-4* (−0.04, *p* = 0.74).

**Figure 2:**
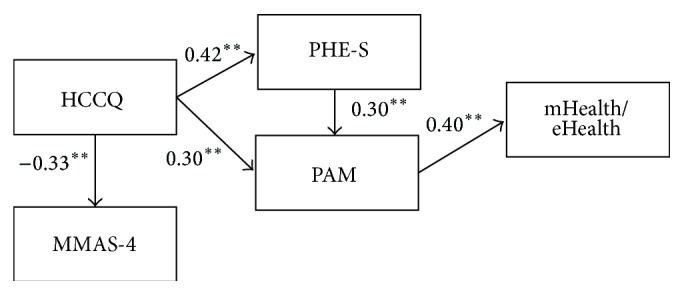
Structural Equation Model 2.

The hypotheses were only partially verified. On the basis of the evaluation of the modification indexes, the correlations, and the estimated paths, a modification of the model was hypothesized and tested. In particular modification indexes suggested to emphasize the direct relationship between* HCCQ* and* MMAS-4* and to delete the relationship between* frequency of mHealth/eHealth use* and* MMAS-4*. The* MMAS-4* resulted consequently from a high level of patients' perceptions about the ability of the healthcare system in supporting their autonomy (*HCCQ*). The* frequency of mHealth/eHealth use* resulting is strongly dependent on the level of patients' activation (*PAM*), but it did not seem to impact on patients' adherence (*MMAS-4*).  Figure 2 shows the final model.

Model 2 presented an acceptable goodness-of-fit. Chi square test was not significant (*χ* ^2^(5) = 7.54, *p* = 0.15). All the goodness-of-fit was satisfactory (RMSEA = 0.07, CFI = 0.90, and SRMR = 0.06). The estimated paths were significant (*p* < 0.001). The adjusted goodness-of-fit (AGFI) was superior to 0.90 (AGFI = 0.901). Overall, model fit indices significantly increased from Model 1 to Model 2.

## 4. Discussion

This study aimed to verify how the perceived ability of the healthcare professionals to support type 2 diabetes patients' autonomy and motivation to self-care initiative might impact on their level of activation and engagement and, consequently, on their adoption of mHealth and eHealth technologies to seek information for managing care. Furthermore, the study aimed to test the mediating role of patient engagement in the relationship between the healthcare professional motivating role and patient activation. Finally, the study explored the impact of mHealth and eHealth technologies use for health information seeking on type 2 diabetes patients' adherence.

Concerning the first two hypotheses, the study confirmed the crucial role of the healthcare professionals in influencing the level of type 2 diabetes patients' engagement and activation, according to other studies on chronic populations [17]. Furthermore, the level of type 2 diabetes patients' activation was confirmed in influencing patients' adoption of mHealth/eHealth technologies to support care management and seek health information [48, 49].

This study showed how the more clinicians are perceived by patients as able to motivate their initiatives towards self-care, the more the patients report higher level of engagement and activation in healthcare processes. Type 2 diabetes patients' perception and assessment of the healthcare professionals' ability to be aligned with their needs and expectations toward care management are, thus, demonstrated to be a crucial antecedent of the patients' ability to take an active role in their care management. The more the healthcare system is perceived as facilitating type 2 diabetes patients' autonomy, the more the patients show higher level of engagement towards their care management. To foster patients engagement in care management means to support the complex psychosocial elaboration of the illness condition and of the new medical requirements that individuals undergo when diagnosed with type 2 diabetes (and/or when new symptoms occur) [14, 50]. Consequently, the role of healthcare professionals appears pivotal in supporting type 2 diabetes patients engagement in adopting healthier lifestyles and gaining higher quality of life [29, 51].

Furthermore, as this study showed, high level of type 2 diabetes patients engagement is predictive of the patients activation in self-management: the more the type 2 diabetes patient is engaged, the more he/she appears able to feel self-confident in assuming a proactive and empowered role in the care process. The huge impact of cognitions and behaviors is well reported in literature [14, 29]. However, patients' engagement is the result of a dynamic synergy among different experiential dimensions: patient engagement, indeed, is not only dependent on knowledge and skills related to the health condition and treatment management. It also implies patients' enactment of an adaptive emotional elaboration and acceptance of the new patient identity and of its consequences on quality of life [14, 22].

The level of type 2 diabetes patients' activation in its turn resulted to be a crucial antecedent of patients' attitude towards the adoption of mHealth and eHealth technologies to seek information for care management. Patients' activation refers to the patients' ability and willingness to directly manage their own health and health care [39]. To seek health and care information through mHealth and eHealth technologies to manage care might be considered as a behavioral manifestation of the patients' willingness of taking a “starring role” in the management of their care [50].

Different studies investigated the potential role of mHealth/eHealth technologies to support patient activation and used the patient activation as a compass to personalize the intervention with promising results [35, 52]. In this sense, our study provides further evidences on a crucial antecedent of patient activation: that is patient engagement. This concept might be useful when developing and delivering technological solutions, which are aligned with the complex emotional elaboration the patient undergoes when dealing with diabetes care and allow them to communicate with their referential health professional [53].

Moreover, our results confirmed the importance of questioning the readiness of the healthcare organization and of its employees in receiving and adopting technological innovations devoted to sustaining better integrated models of care [54, 55]. Implicit values and practices rooted into the organizational culture might play the role of enhancers or inhibitors of such organizational innovation. Relational, psychological, and pragmatic implications of eHealth and mHealth should be considered when planning and delivering such interventions in order to maximize their clinical and organizational effectiveness. Healthcare professionals' education oriented to uncovering of clinicians' experiential knowledge and attitudes towards patients' engagement should be a priority in this changing scenario [56].

Finally, it is interesting to note that the last hypothesis of this study was not confirmed. The level of patients' adherence was not proved to be directly dependent on the frequency of mHealth and eHealth adoption to seek information for type 2 diabetes care management, thus demonstrating that this is still a controversial topic according to other studies [57]. In this sense, spontaneous behaviors of information seeking through mHealth and eHealth sources are not an indication of greater patients' adherence. Health information obtained through online sources has been widely debated for their inaccurate and misleading nature which can lead to ineffective self-care regimens if not properly sustained by healthcare professionals [58]. Furthermore, the ability of mHealth or eHealth to foster type 2 diabetes patients' adherence might be dependent on the characteristics of the intervention and of the specific tools employed in it; mHealth and eHealth tools for information seeking probably need tailored and multiple strategies to promote adherence [57]. Patients' adherence resulted, on the contrary, from direct function of the healthcare professionals' perceived ability to support patients' autonomy and motivation towards their diabetes care. This result appears particularly interesting because it is a further empirical confirmation of the crucial role played by the healthcare organization and by its employees to enable the success of clinical interventions. Indeed, healthcare professionals seem to have a vicarious role in the proper use of health information and in the activation of patients towards managing their health and, consequently, in patients' adherence. Different studies confirmed that the quality of the relationship between healthcare professionals and patients is a crucial factor for improving the adherence of patients [59, 60]. Our results suggest the importance of supporting the introduction of new technological tools to innovate healthcare processes with a deep understanding of the psychosocial, relational, and pragmatic implication of such innovation: only “taking on board” the human resources implied in this organizational change, the challenge of innovating care process in an effective integrated model can be successful [61, 62]. Healthcare professionals, in particular, need to be accompanied to understand and accept the value of such tools to improve their ability to follow and treat their type 2 diabetes patients. Healthcare professionals are the enablers, from patients' perspective, of the mHealth or eHealth interventions' clinical potentials; they are perceived as the legitimators of the active role of the patient in the care process [17] and thus of the possibility to adopt new technologies within the type 2 diabetes care pathway within a shared decision making process [63].

Therefore, mHealth or eHealth initiatives for type 2 diabetes care should be designed and delivered having in mind the goal of sustaining the engagement of the different stakeholders implied in the healthcare process (i.e., the patients, their lay caregivers but also their healthcare professionals both inside and outside the hospital) [11, 14, 38]. This goal could be achieved by assuming a psychosocial and organizational view of the different level of needs and expectations towards the care process (and its innovation) carried out by the different stakeholders: to fail in this consideration may result in psychosocial and relational hindrances to the process of adoption of mHealth or eHealth and thus to their clinical effectiveness. This could also have an impact on the success of integrated care models featuring the adoption of new technologies [12].

*Limitations*. Although the results of our study appear interesting to cast light on the complex psychosocial and organizational dimensions implied in sustaining patient engagement and the adoption of mHealth or eHealth for seeking information for type 2 diabetes care in integrated care models, some limitations have to be considered. Firstly, the study was carried out on a fairly small sample of Italian patients. However the sample features were enough to allow the robustness of the conducted statistical analysis. Furthermore, the sample of patients included in our study is not representative of the Italian type 2 diabetes population. However, we used it only to explore the relationships of the variables under analysis and not for an estimation of their dimensions: based on these considerations full representativeness is not necessarily required [64, 65]. Furthermore, our study was not conceived as an effectiveness evaluation of a real mHealth or eHealth intervention, but it took into account the spontaneous behaviors of patients when adopting mHealth or eHealth technologies to seek information for type 2 diabetes care management. This may be envisaged as a limitation because it does not allow the researcher to understand what technological and organizational characteristic of a mHealth or eHealth intervention may impact on patients' engagement and activation and on their adherence to treatment. Results should be interpreted with caution because of the explorative nature of this study. Furthermore, we only measured the frequency of spontaneous behaviors of mHealth and eHealth use to seek information for diabetes care instead of measuring also type of technologies adopted or type of information searched.

However, this analysis has the value of offering some precious insights into the patients' spontaneous attitudes and behaviors in a natural setting and should be considered as a “baseline” evidence of the general approach of patients to mHealth or eHealth and of the psychosocial and organizational dynamics that may impact on their effectiveness [66].

## 5. Conclusions

Type 2 diabetes requires a long-term approach to care and the good synergy between hospitals and primary care resources. To address this requirement, to “give back” an active role to patients in managing their health is crucial. mHealth/eHealth interventions for type 2 diabetes care are considered as an effective strategy to improve type 2 patients' empowerment and clinical outcomes. Moreover they are demenstrated to be powerful in enhancing patients-doctors communication, in fostering patients' satisfaction with care and in making healthcare cost-effective. However, in order to be effective, the introduction of such technological interventions needs to be supported by the reference healthcare professionals, who should legitimize the intervention process and sustain the autonomous initiative of the type 2 diabetes patients throughout it.

From this perspective, our study confirmed the important role of healthcare professionals' ability to foster type 2 diabetes patients' autonomy in enhancing their activation and engagement towards self-management, this being a precursor of patients' attitude to the use of mHealth/eHealth technologies. Furthermore, our study well highlighted how patient engagement, defined as a multidimensional psychosocial process resulting from the conjoint cognitive, emotional, and behavioral enactment of individuals toward their health conditions and their management [15, 17, 38], is a pivotal precursor of patient activation towards self-management and thus towards patients' use of new technological interventions. This finding is relevant and opens insights into the psychosocial and relational antecedent of patients' activation in self-management. The function of patients' activation in guaranteeing improved clinical outcomes, better patients' satisfaction towards healthcare, and reduced costs in services delivery has been demonstrated by several studies [67–69]. However, till now, still little is known about the factors that may support the increase of patients' activation [70]. This study, by focusing on type 2 diabetes patients, offers an important theoretical and pragmatic contribution by demonstrating the role of patient engagement in determining the level of patients' behavioral activation and self-confidence in type 2 diabetes care management.

Finally, the indirect relationship that our study showed between the frequency of mHealth/eHealth use and the level of type 2 diabetes patients' adherence, although it needs further confirmation, opens the door to interesting debate about how new technologies can be effectively designed in order to improve adherence. Too often, the debate about new mHealth/eHealth interventions for sustaining patient engagement in type 2 diabetes care management has been primarily focused on the technological (“hard”) features of such interventions [71]. The psychosocial and organizational (“soft”) aspects may mediate the effectiveness of mHealth and eHealth interventions and, consequently, deserve an enhanced attention, as an important complement of the analysis of the “hard” determinants of such interventions effectiveness [72].

## Figures and Tables

**Table 1:** Characteristics of the sample.

Sociodemographic characteristics	
Age (years)	M = 58.3; DS = 12.4
Gender (% female)	31.2
Disease duration	M = 14.4; DS = 11.1
Marital status (%)	
Never married	7.5
Married	79.5
Divorced	10.8
Widowed	2.2
Employment (%)	
Employed	43.0
Retired	44.0
Housewife	3.2
Student	2.2
Unemployed	5.4
Other	2.2
Education (%)	
Elementary school	5.4
Junior high school	14.0
High school	50.5
College education	23.7
Ph.D. or M.S. degree	6.4

Psychometric measures	
PHE-S	Median = 3 (range 1–4); entropy = 0.89; ordinal alpha = 0.82
PAM	M = 66.8 (range 0–100); DS = 18.3; Cronbach's alpha = 0.93
MMAS-4	M = 1.3 (range 0–4); DS = 1.3; Cronbach's alpha = 0.81
HCCQ	M = 66.8 (range 13–91); DS = 15.1; Cronbach's alpha = 0.92

**Table 2:** Frequency of mHealth/eHealth use.

I usually use internet or mobile devices to seek information for managing my care (%)	
Never	14.0
Almost never	5.3
Occasionally	5.3
Sometimes	19.4
Often	17.2
Almost always	19.4
Always	19.4

**Table 3:** Linear correlations coefficients between psychometric measures and frequency of mHealth/eHealth use.

	HCCQ	PHE-S	PAM	MMAS-4	mHealth/eHealth
HCCQ	—	0.356^*∗∗*^	0.406^*∗∗*^	−0.315^*∗∗*^	0.292^*∗∗*^
PHE-S		—	0.428^*∗∗*^	−0.244^*∗*^	0.034
PAM			—	−0.222^*∗*^	0.373^*∗∗*^
MMAS-4				—	−0.090
mHealth/eHealth					—

^*∗*^ *p* < 0.05; ^*∗∗*^ *p* < 0.01.
