# Corrigendum to “The Motivating Function of Healthcare Professional in eHealth and mHealth Interventions for Type 2 Diabetes Patients and the Mediating Role of Patient Engagement”

**DOI:** 10.1155/2019/9246276

**Published:** 2019-11-20

**Authors:** Guendalina Graffigna, Serena Barello, Andrea Bonanomi, Julia Menichetti

**Affiliations:** ^1^Department of Psychology, Università Cattolica del Sacro Cuore, Largo A. Gemelli 1, 20123 Milan, Italy; ^2^Department of Statistical Sciences, Università Cattolica del Sacro Cuore, Largo A. Gemelli 1, 20123 Milan, Italy

In the article titled “The Motivating Function of Healthcare Professional in eHealth and mHealth Interventions for Type 2 Diabetes Patients and the Mediating Role of Patient Engagement” [1], the Morisky Medication Adherence Scales (MMAS-4) was wrongly mentioned. The correct scale used in this work is the Morisky Green Levine Scale (MGLS). The latter is an older, 4-item scale that is different from the MMAS-4, in particular in the formulation of the second item of the scale. Therefore, “Morisky Medication Adherence Scale” and “MMAS-4” should be corrected to “Morisky Green Levine Scale” and “MGLS” throughout the entire article.
